# Genotype-Specific Changes in Vitamin B_**6**_ Content and the PDX Family in Potato

**DOI:** 10.1155/2013/389723

**Published:** 2013-07-18

**Authors:** Sutton Mooney, Liyuan Chen, Christina Kühn, Roy Navarre, N. Richard Knowles, Hanjo Hellmann

**Affiliations:** ^1^Washington State University, Pullman, WA 99164, USA; ^2^Humboldt University Berlin, 10115 Berlin, Germany; ^3^USDA-Agricultural Research Service, Prosser, WA 99350, USA

## Abstract

Vitamin B_6_ is one of the most versatile cofactors in plants and an essential phytonutrient in the human diet that benefits a variety of human health aspects. Although biosynthesis of the vitamin has been well resolved in recent years, the main research is currently based on *Arabidopsis thaliana* with very little work done on major crop plants. Here we provide the first report on interactions and expression profiles of *PDX* genes for vitamin B_6_ biosynthesis in potato and how vitamin B_6_ content varies in tubers of different genotypes. The results demonstrate that potato is an excellent resource for this vitamin and that strong natural variation in vitamin B_6_ content among the tested cultivars indicates high potential to fortify vitamin B_6_ nutrition in potato-based foods.

## 1. Introduction

Vitamin B_6_ (vitB_6_) is one of the most versatile cofactors found in living organisms. This generic term comprises three different compounds: pyridoxine (PN), pyridoxal (PL), and pyridoxamine (PN). When phosphorylated at their 5′ position (e.g., pyridoxal-5′-phosphate (PLP)) these compounds can serve as active cofactors in various biochemical reactions such as transaminations, decarboxylations, and racemizations that function primarily in amino acid metabolism [1]. However, the vitamin also plays a role in fatty acid and carbohydrate turnover [1]. In addition, B_6_ vitamers function as potent antioxidants that help protect plants against abiotic stress conditions such as salt, high light, or drought, and are used by some pathogens as a neutralizing agent against photosensitizing toxins [2–4]. Due to its central role in general plant development and physiology, it is not surprising that mutants affected in vitB_6_ biosynthesis display diverse defects including, for example, reduced chlorophyll, late flowering phenotypes, increased stress sensitivities, and aberrant embryogenesis [5–10]. Because the basic role of vitB_6_ is conserved among humans and plants, the vitamin has also been implicated in many different health-related aspects ranging from diabetes and neurological disorders to cardiovascular diseases [11]. Plants, in contrast to humans, can synthesize vitB_6_ *de novo* and thus represent an excellent dietary source of this essential compound. The biosynthesis requires the concerted activities of the PDX1 (pyridoxal biosynthesis 1) and PDX2 proteins that form a large multimeric complex to synthesize PLP from glutamine, a pentose-phosphate (ribose 5′-phosphate or ribulose 5′-phosphate) and a triose-phosphate (dihydroxyacetone phosphate or glyceraldehyde 3′-phosphate) [8, 12]. 

Potato is one of the major food crops worldwide and the leading vegetable crop in the United States, with a production of 427,406 million cwt in the USA in 2011 (http://usda01.library.cornell.edu/usda/current/CropProdSu/CropProdSu-01-11-2013.pdf). Although potato is an excellent dietary source of vitamins and minerals, it is also denigrated for its high glycemic index and a source for high fat and acrylamide containing food due to specific food processing steps [13–15]. Little is known about variations of this critical phytonutrient in different genotypes and how the biosynthetic pathway functions in this plant.

Here we describe the PDX family in potato based on yeast-2-hybrid screens, their expression in different tissues, and how vitB_6_ contents vary in tubers among different potato varieties and after prolonged storage. The results indicate that the PDX family is highly conserved among different plant species, that potato is a very good resource for the vitamin, and that vitB_6_ content varies substantially among the tested genotypes. There is thus great potential for improving potato further through increasing the content of this specific phytonutrient, by either breeding or genetic manipulation to fortify the B_6_ vitamer as a healthy food resource for human nutrition.

## 2. Material and Methods

### 2.1. Plant Material

The potato cultivar Defender was grown in a culture chamber setup for long-day conditions (18 h : 6 h light : dark; 20°C; 60% humidity) and used for qRT-PCR analysis, and all other samples came from a local supermarket or from field grown plants. All field grown plants were grown at the Washington State University Irrigated Agriculture Research Unit at Othello, WA, USA (46°47.277′N Lat., 119°2.680′W Long.) under standard commercial conditions. Baby potatoes analyzed in this work are defined as immature tubers harvested from young plants, typically 60–80 days after planting (DAP). Mature tubers were harvested from the same field at the end of the growing season, typically 160–179 DAP. Defender and Russet tubers used as samples for studies of the effects of storage time and temperature on vitB_6_ and reducing sugar (Glc + Fru) content, respectively, were grown as described by [16] and harvested in October 11, 2005 (181 days after planting). The harvested tubers were sorted according to weight, and tubers ranging from 170 g to 284 g were placed in storage (12°C, 95% RH) to wound heal for 11 days. Tubers of each cultivar were then stored at 6.7 and 8.9°C (95% RH) for 227 days (until May 26, 2006). Reducing sugar (Glc + Fru) and vitB_6_ levels were quantified in lyophilized samples of tuber tissue. Four replicates of five tubers each were sampled at 11 and 227 DAH. A central slice (approximately 1.5 mm thick, periderm attached) along the apical to basal axis from each of the five tubers was consolidated for each replicate, collectively frozen at −80°C and lyophilized. The dried tissue was ground with a mortar and pestle and sieved through a 60 mesh (0.246 mm) screen in preparation for vitB_6_ (see below) and reducing sugar determinations. Glucose and fructose were estimated according to a microplate modification of [17, 18] as described in [16]. The stoichiometric reduction of NADP as each hexose is converted to 6-phosphogluconate was monitored at *A*_340_, and quantitation was based on Glc and Fru standard curves.

### 2.2. Yeast-Two-Hybrid Screens

Y2H screens were performed on a mixed potato tissue cDNA library described in [19], following standard procedures as previously described in [20]. The library was in the beginning shuffled into the Gateway-compatible *pACT2* (CLONTECH, Palo Alto, CA, USA; GenBank accession no. U29899) prey vector [21]. The corresponding Arabidopsis baits AtPDX1.3 and AtPDX2 were cloned into *pBTM116-D9*. All primary positive colonies from the initial screen were picked and first rescreened on medium containing 5 mM 3-amino-1,2,4-triazole (3AT) to reduce the potential of analyzing autoactivators. Growing colonies under these conditions are referred to as “secondary positives.” DNA was isolated from 10 secondary positive yeast colonies, retransformed into yeast, and verified for positive interactions with either PDX1.3 or PDX2, respectively, as well as lack of auto-activation in the presence of an empty *pBTM116-D9* vector. DNAs for clones that were verified to be positive were subject to sequencing. Based on gained sequence information, specific primers were designed for screening of the remaining secondary positives. All secondary positives for which no PCR product was gained underwent the same selection process of DNA as described above (isolation, retransformation, verifying interaction of auto-activation, and sequencing) to design new primer sets. This procedure was pursued till all secondary positive had been classified as summarized in Table 1. Primers used in the screen are shown in Supplementary Table  2 (see Supplementary Material available online at http://dx.doi.org/10.1155/2013/389723).

### 2.3. qRT-PCR Analysis

RNA isolation and first strand cDNA synthesis was done using an Isolate RNA Kit (Bioline USA Inc., MA) and a High Capacity cDNA Reverse Transcription Kit (Applied Biosystems, MY, USA), respectively. Quantitative RT-PCR reactions were performed following the protocol of 95°C for 7 mins, 40 cycles of 95°C for 15 secs, and 60°C for 1 min, on a 7500 Fast Real-Time PCR System (Applied Biosystems, NY, USA). *ACTIN2* gene in potato was selected as reference gene in qRT-PCR for data modification. 50 ng of cDNA from different tissues of potato plants was used for template of each reaction. Primers used for qRT-PCR are shown in Supplementary Table 2.

### 2.4. Yeast Bioassay

VitB_6_ extraction and measurements of total vitB_6_ content from fresh and lyophilized tuber samples were done by a yeast bioassay, adapted from a protocol described in [9], and a vitB_6_ auxotrophic strain, ATCC9080. Data shown are averages of four biological replicates, each of which was in addition subject to three technical replicates. Yeast growth rates were followed at OD_540_ and used to determine total vitB_6_ content based on comparison to a standard curve.

## 3. Results

### 3.1. Screening of Y2H Library Yields Potato PDX1 Proteins and Novel Interactors

**Figure 1:**
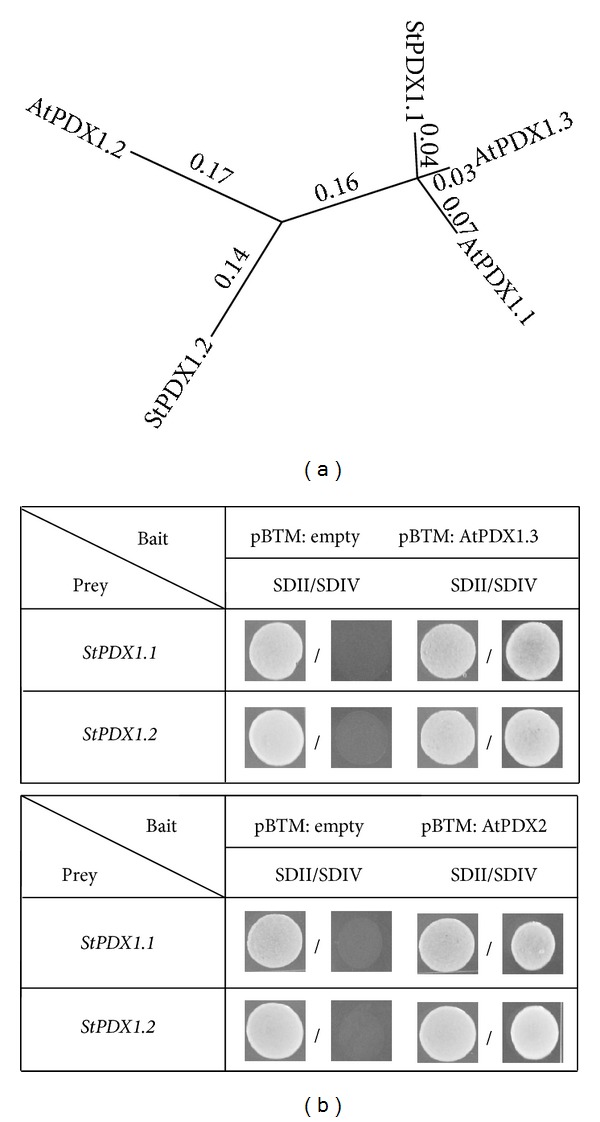
Phylogenetic relationship and interaction of potato and *Arabidopsis* PDX proteins. (a) An unrooted phylogenetic tree comparing Arabidopsis and potato PDX1 proteins. Proteins were aligned through Clustal W in a pairwise order.  (b) Y2H results for screens with AtPDX1.3 (upper half) or AtPDX2 (lower half) as baits, respectively. SDII: selection medium for transformation with bait (*pBTM116-D9*) and prey (*pACT2*) plasmids supplemented with leucine and histidine; SDIV: selection medium for interaction studies without leucine and histidine supplements. Photos were taken from single spots.

**Figure 2:**
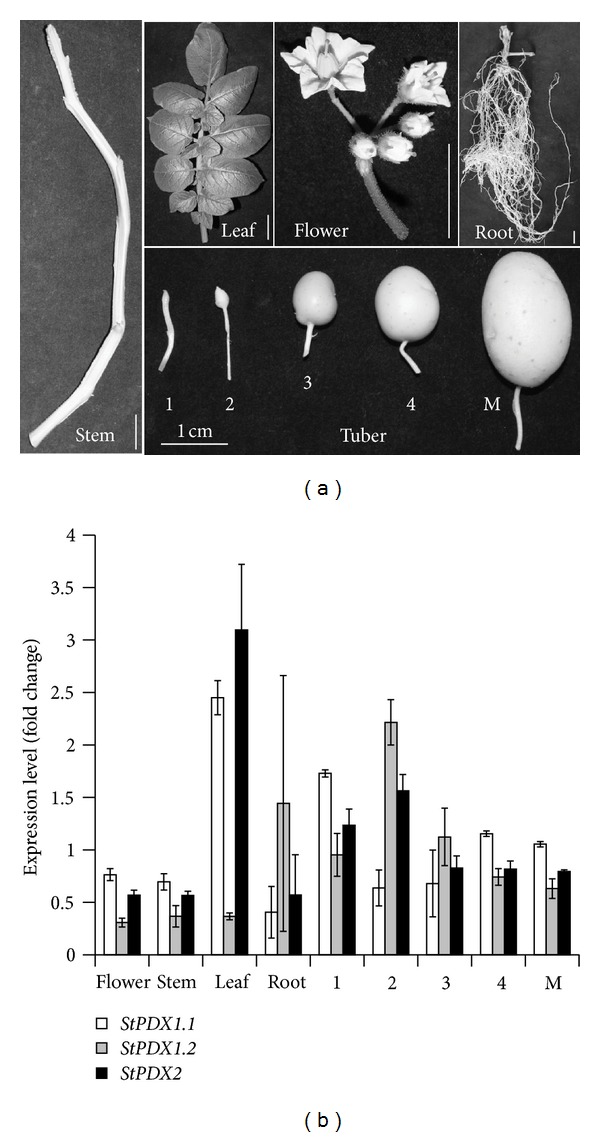
qRT-PCR expression analysis of *StPDX1* and *StPDX2* genes in different potato tissues. (a) Pictures taken of tissues used for qRT-PCR analysis. (b) qRT-PCR analysis for StPDX1.1, StPDX1.2, and StPDX2. 1 to 4 are the different developmental tuber stages; M: mature. In this and all subsequent figures, error bars indicate the value of standard deviation, while single asterisks indicate a value of *P* < 0.05, and two asterisks a value of *P* < 0.01 (one-way ANOVA; *P* values were calculated in relation to the lowest value in each graph). Defender, from tissue culture, 12/23/2009–2/4/2010 (100 d).

To identify expressed members of the potato PDX family, we performed two yeast-two-hybrid (Y2H) screens on a potato cDNA library generated from mixed tissues (source and sink leaf, root, stem, flower, petiole, and tuber; [19]). We decided to use *Arabidopsis thaliana* PDX proteins as baits since their function has been studied in great detail in the last years. In addition, their sequence and function appear to be conserved not only between Arabidopsis and *Ginkgo biloba*, but also in bacteria, making it highly likely that this would also be seen in potato [22]. One screen was done with Arabidopsis AtPDX1.3/At5g01410, and another with Arabidopsis AtPDX2/At5g60540. Through this combinatorial approach we anticipated to gain the corresponding expressed StPDX orthologs from potato, and potentially some novel interacting candidates. In total 182 and 304 primary positive clones were seen from the AtPDX1.3 and AtPDX2 screens, respectively. From those DNA was isolated and retransformed into the yeast, which resulted in 44 and 187 secondary positive clones. These remaining clones were further analyzed through sequencing and PCR to gain a better idea of the nature of these clones. Two different *StPDX1* clones were identified in either screen, PGSC0003DMT400078045 and PGSC0003DMT400014535 that showed similarities to Arabidopsis *AtPDX1* genes (Table 1). Phylogenetic comparison with Arabidopsis AtPDX1 proteins showed that PGSC0003DMT400078045 was most closely related to AtPDX1.1 (87.7% identity) and AtPDX1.3 (91.9% identity) (Figure 2(b)) and was named StPDX1.1. In contrast, PGSC0003DMT400014535 showed greater similarities to AtPDX1.2 (69% identity to AtPDX1.2 and 65% and 63% identity to AtPDX1.3 and AtPDX1.1, resp.), and was named accordingly StPDX1.2 (Figure 1(b)). While we found a few *non-PDX* encoding genes in either screen (Table 1; Supplementary Table 1), no *StPDX2* was present among the analyzed clones. This was unexpected since a functional PLP synthase depends on the concerted activities of PDX1 and PDX2 proteins [8]. In addition, publicly available sequence information predicts the presence of a *StPDX2* gene (*PGSC0003DMT400020828*), which shows 75% identity on the protein level to AtPDX2. 

### 3.2. *StPDX* Genes Are Expressed in All Potato Tissues

To further analyze the identified *StPDX1* clones, we performed qRT-PCR analyses on different potato tissues shown in Figure 2(a). We also included the annotated *StPDX2* to investigate whether and to what extent the gene is expressed in potato. As shown in Figure 2(b), all three *PDX* genes are expressed in all tested tissues. *StPDX1.3* and *StPDX2* show the highest levels of expression in leaf, while expression of *StPDX1.2* is highest in sink tissues, including root and early tuber development. It was noteworthy that *StPDX2* and *StPDX1.3* showed high levels of expression preferentially in early tuber development (Figure 2(b)). Overall the data confirm that *StPDX1.2 *and* StPDX1.3* are expressed in potato and demonstrate that this is also seen for the annotated *StPDX2* gene.

### 3.3. VitB_6_ Content Varies Significantly among Different Genotypes and under Long-Term Storage Conditions

**Figure 3:**
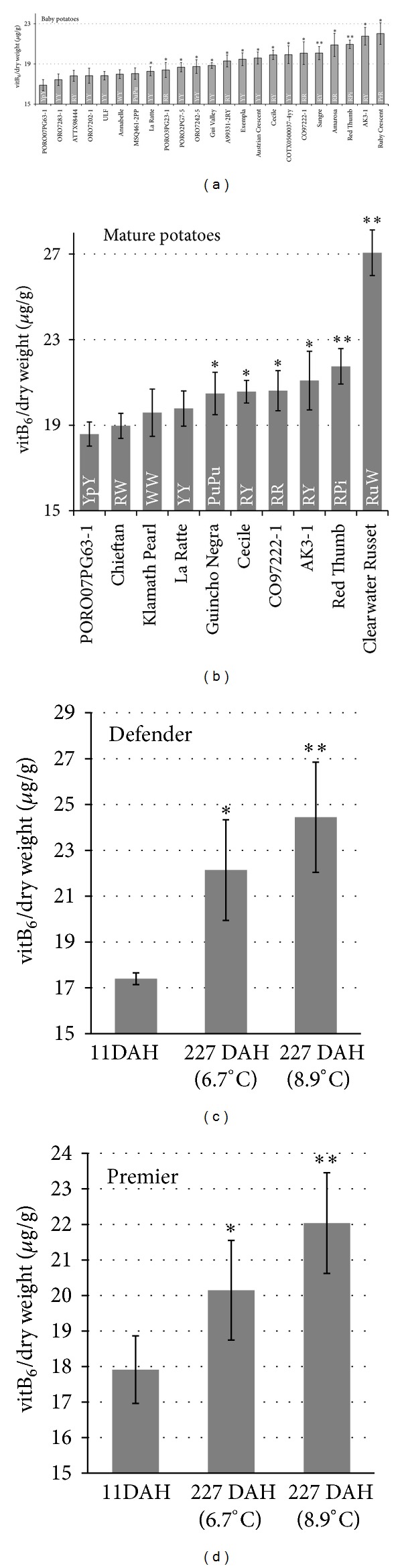
Total vitB_6_ content in potato tubers based on a yeast bioassay. (a) Extracts from lyophilized baby potato tubers. (b) Mature potatoes, and ((c), (d)) vitB_6_ content in two cultivars after long-term, low-temperature storage. Letters indicate skin (first capital letter) and flesh color (second capital letter). Pi: pink; Pu: purple; Pr: pink red; R: red; Ru: Russet, W: white; Y: yellow; Yp: Yellow with pink eyes; Yr: yellow with red eyes.

VitB_6_ is an essential phytonutrient in the human diet, and potato is a major food crop. We were curious if there were major differences in vitB_6_ content based on cultivar. Our first approach was to analyze tubers of four different potato varieties bought in a supermarket, Russet, French Fingerling, Austrian Crescent, and Purple Peruvian. The measurements showed that a Russet potato of 150 g weight contains 180 *μ*g vitB_6_ on average, while a fingerling specialty potato, like the Purple Peruvian, has 240 *μ*g vitB_6_ when consumed at equivalent amounts (Supplementary Figure 2). The data indicated that coloration of the potato may correlate with increased vitB_6_ content and emphasized a remarkable difference present in the four genotypes. To verify these observations we expanded our test to 29 additional cultivars (Figure 3). Since expression of *StPDX* genes was highest in developing tubers we focused our analysis on young tubers. As shown in Figure 3(a), analysis of 23 different varieties of baby potatoes showed a variation in total vitB_6_ content that ranged from 16.9 *μ*g vitB_6_/g dry weight (PORO07PG63-1) to 22 *μ*g vitB_6_/g dry weight (Ruby Crescent), a 30% increase from the lowest to highest level. Analysis of mature tubers from 10 different cultivars showed that vitB_6_ level also varied significantly among the different genotypes with the lowest being again PORO07PG63-1 (18.6 *μ*g vitB_6_/g dry weight), with surprisingly high levels for Clearwater Russet (27 *μ*g vitB_6_/g dry weight). Samples that allowed comparison between baby and mature potatoes used in this analysis (PORO07PG63-1, La Ratte, Cecile, CO97222-1, Red Thumb, and AK3-1) showed no consistent effects of tuber maturity, indicating that vitB_6_ concentrations are likely comparably throughout tuber growth. In addition, the analysis of a broader range of cultivars did not support the notion that vitB_6_ levels correlate with a specific skin or flesh color. Finally, we investigated changes of vitB_6_ content under long-term, low-temperature storage for two potato cultivars, Defender and Premier Russet (Figures 3(c) and 3(d)). For both cultivars, vitB_6_ concentrations increased significantly over a 227-day storage period at either 6.7 or 8.9°C, and the increases were greatest, averaging 42% (Defender) and 23% (Premier), at the highest temperature (Figures 3(c) and 3(d)). 

## 4. Discussion

The current work identified two potato PDX1 proteins as interactors of Arabidopsis PDX1 and PDX2 proteins. In addition, the work provides first insights on tissue-specific expression profiles, and how vitB_6_ content of tubers differs among various cultivars and over a 7.5-month storage period at temperatures consistent with those used in the industry.

The finding of only two different StPDX1 members in the Y2H screen with either bait is in agreement with the blast searches using Arabidopsis and potato PDX1 sequences against the available potato genome database (http://solanaceae.plantbiology.msu.edu/). This currently indicates that potato likely encodes for only two *StPDX1* genes, in contrast to Arabidopsis that has three *AtPDX1* family members. It was surprising that no PDX2 was found since a functional PLP synthase requires the activity of PDX2 proteins, because the potato genome encodes for a PDX2 protein, and we can demonstrate that the corresponding gene is expressed. The reason for this absence of StPDX2 in the screen remains unclear but may be due to sequence differences among Arabidopsis and potato PDX2s that affect protein-protein interaction, since both proteins only share 69% identity. In this context it is also noteworthy that StPDX1.2 interacted with AtPDX2, which is remarkable since AtPDX1.2, to which StPDX1.2 is most closely related, is unable to assemble with AtPDX2 [6]. This again indicates that sequence variances between Arabidopsis and potato PDX proteins are responsible for this difference in interaction behavior, since AtPDX1.2 and StPDX1.2 are also only 69% identical. It will be interesting to determine in future experiments whether the potato StPDX2 is also unable to assemble with StPDX1.2 as observed for the Arabidopsis orthologs.

An interesting finding that may be worth pursuing is the novel interactions found in the two screens (Supplementary Table 1, Supplementary Figure 1). Although these interactions need to be verified for the potato StPDX proteins, they may suggest additional pathways or regulatory mechanisms by which StPDX activity is controlled in potato. These interactions may have a regulatory role on the PLP synthase or lead to a novel complex with a function distinct from vitB_6_ biosynthesis (Supplementary Table 1).

VitB_6_ plays a major role in various cellular reactions and confers many health benefits for humans, which may in part be attributed to its antioxidant capabilities [11, 12, 23]. The Recommended Dietary Allowance from the American National Institute of Health (NIH) for vitB_6_ is 2 mg/day for adults, and given that there is a possibility of vitB_6_ malnutrition, as earlier reported for the USA [24], our data indicate that potato is a good source of this vitamin, regardless of tuber maturity or coloration. The significant variation observed among the tested genotypes indicates high potential for further increasing vitB_6_ content of potato through directed breeding efforts. 

Finally, starch breakdown and sweetening of tubers during long-term storage and under low-temperature conditions (cold sweetening) are undesirable for the potato processing industry due to the synthesis of dark-colored Maillard reaction products during frying. These products are a result of heat-induced nonenzymatic reactions between reducing sugars (Glc and Fru) and free amino acids to cause browning and acrylamide formation in fried potato products [15, 25]. The initial steps in starch breakdown depend in part on the activity of *α*-glucan phosphorylase, a PLP-dependent enzyme, which mediates the release of glucose-1-phosphate from *α*-1,4-linked glucan chains [26, 27]. 

Defender [28] is susceptible to sweetening during storage while Premier Russet is highly resistant [28–30]. Reducing sugar (Glc plus Fru) concentrations of the Defender tubers depicted in Figure 3(c) increased from 5.94 ± 0.98 mg/g dry wt at 11 DAH to 30.6 ± 1.8 and 26.1 ± 1.3 mg/g dry wt when stored for 227 days at 6.7 and 8.9°C, respectively. In contrast, the Premier Russet tubers depicted in Figure 3(d) contained 1.81 ± 0.37 mg/g dry wt reducing sugar at 11 DAH, and levels increased to only 11.9 ± 1.4 and 4.88 ± 0.61 mg/g dry wt when stored at 6.7 and 8.9°C, respectively, reflecting the inherently greater resistance to sweetening in this cultivar. These genotype-dependent differences in reducing sugar buildup over the 227-day storage period resulted in unacceptably dark processed fries for Defender, but fries from Premier Russet remained acceptably light (data not shown). The greater increase in vitB_6_ content of Defender from 11 to 227 DAH was thus consistent with the higher levels of sweetening (and starch catabolism) in this cultivar. However, while the increases in vitB_6_ may have a role in modulating the activity of *α*-glucan phosphorylase, vitB_6_ levels were not directly correlated with the temperature-dependent buildup in reducing sugars in either cultivar. Further work is warranted to establish whether vitB_6_ correlates with *α*-glucan phosphorylase activity and to elucidate its potential involvement in the cold-induced and genotype-dependent differences in sweetening metabolism reported here.

It is noteworthy that vitB_6_ content of tubers increased postharvest, particularly when considering that vitamin C declined 62% in Defender and 51% in Premier Russet over a similar storage period [31]. Hence, with regard to vitB_6_, the tubers became more nutritious with time in storage. While both cultivars had approximately the same vitB_6_ content 11 DAH (ca. 17.6 *μ*g/g dry wt), Defender tubers contained 10% higher levels than Premier tubers by 227 DAH. These data suggest that the capacity for postharvest increases in vitB_6_ content of tubers may be genotype dependent, and further work is warranted to determine the heritability of this potentially important trait for breeding programs. 

## 5. Conclusions

The current work emphasizes that potato is an excellent nutritional resource for vitB_6_ with great potential for fortifying vitamin B_6_ nutrition. The biosynthetic pathway is conserved among plants, and the corresponding genes are expressed in all tested tissues. The identification of new interactors is interesting and may open up new research directions with work related to PDX function in the future. 

## Supplementary Material

Supplementary Table 1 summarizes findings for non-PDX proteins found in the performed Y2H screens on a mixed cDNA library containing samples from different potato tissues. The actual test for interactions of these proteins with PDX members is shown in Supplementary Figure 1. Major variation in total vitB6 content is present in fresh potato varieties purchased from a local supermarket (Supplementary Figure 2). Supplementary Table 2 provides an overview of primers used in this work.

## Figures and Tables

**Table 1:** Results of Y2H screening.

Bait/prey	StPDX1.2	StPDX1.3	Non-PDX
AtPDX1.3	7	29	6
AtPDX2	33	146	8

## References

[1] Mooney S, Leuendorf J-E, Hendrickson C, Hellmann H (2009). Vitamin B6: a long known compound of surprising complexity. *Molecules*.

[2] Daub ME, Ehrenshaft M (2000). The photoactivated Cercospora toxin cercosporin: contributions to plant disease and fundamental biology. *Annual Review of Phytopathology*.

[3] Bilski P, Li MY, Ehrenshaft M, Daub ME, Chignell CF (2000). Vitamin B6 (pyridoxine) and its derivatives are efficient singlet oxygen quenchers and potential fungal antioxidants. *Photochemistry and Photobiology*.

[4] Ehrenshaft M, Bilski P, Li M, Chignell CF, Daub ME (1999). A highly conserved sequence is a novel gene involved in *de novo* vitamin B6 biosynthesis. *Proceedings of the National Academy of Sciences of the United States of America*.

[5] Leuendorf JE, Osorio S, Szewczyk A, Fernie AR, Hellmann H (2010). Complex assembly and metabolic profiling of *Arabidopsis thaliana* plants overexpressing vitamin B6 biosynthesis proteins. *Molecular Plant*.

[6] Wagner S, Bernhardt A, Leuendorf JE (2006). Analysis of the *Arabidopsis rsr4-1/pdx1-3* mutant reveals the critical function of the PDX1 protein family in metabolism, development, and vitamin B6 biosynthesis. *Plant Cell*.

[7] Titiz O, Tambasco-Studart M, Warzych E (2006). PDX1 is essential for vitamin B6 biosynthesis, development and stress tolerance in *Arabidopsis*. *Plant Journal*.

[8] Tambasco-Studart M, Titiz O, Raschle T, Forster G, Amrhein N, Fitzpatrick TB (2005). Vitamin B6 biosynthesis in higher plants. *Proceedings of the National Academy of Sciences of the United States of America*.

[9] González E, Danehower D, Daub ME (2007). Vitamer levels, stress response, enzyme activity, and gene regulation of Arabidopsis lines mutant in the pyridoxine/pyridoxamine 5′-phosphate oxidase (*PDX3*) and the pyridoxal kinase (*SOS4*) genes involved in the vitamin B6 salvage pathway. *Plant Physiology*.

[10] Denslow SA, Rueschhoff EE, Daub ME (2007). Regulation of the *Arabidopsis thaliana* vitamin B6 biosynthesis genes by abiotic stress. *Plant Physiology and Biochemistry*.

[11] Hellmann H, Mooney S (2010). Vitamin B6: a molecule for human health?. *Molecules*.

[12] Fitzpatrick TB, Basset GJC, Borel P (2012). Vitamin deficiencies in humans: can plant science help?. *Plant Cell*.

[13] Fernandes G, Velangi A, Wolever TMS (2005). Glycemic index of potatoes commonly consumed in North America. *Journal of the American Dietetic Association*.

[14] Majcher M, Jeleń HH (2007). Acrylamide formation in low-fat potato snacks and its correlation with colour development. *Food Additives and Contaminants*.

[15] Amrein TM, Limacher A, Conde-Petit B, Amadò R, Escher F (2006). Influence of thermal processing conditions on acrylamide generation and browning in a potato model system. *Journal of Agricultural and Food Chemistry*.

[16] Knowles NR, Driskill EP, Knowles LO (2009). Sweetening responses of potato tubers of different maturity to conventional and non-conventional storage temperature regimes. *Postharvest Biology and Technology*.

[17] Bergmeyer HU, Bernt E, Schmidt F, Stork H (1974). *D-Glucose: Determination with Hexokinase and Glucose-fl-Phosphate Dehydrogenase*.

[18] Bernt E, Bergmeyer HU (1974). *D-Fructose*.

[19] Krügel U, He H-X, Gier K (2012). The potato sucrose transporter StSUT1 interacts with a DRM-associated protein disulfide isomerase. *Molecular Plant*.

[20] Weber H, Hellmann H (2009). *Arabidopsis thaliana* BTB/POZ-MATH proteins interact with members of the ERF/AP2 transcription factor family. *FEBS Journal*.

[21] Weber H, Bernhardt A, Dieterle M (2005). Arabidopsis AtCUL3a and AtCUL3b form complexes with members of the BTB/POZ-MATH protein family. *Plant Physiology*.

[22] Leuendorf JE, Genau A, Szewczyk A (2008). The Pdx1 family is structurally and functionally conserved between *Arabidopsis thaliana* and *Ginkgo biloba*. *FEBS Journal*.

[23] Denslow SA, Walls AA, Daub ME (2005). Regulation of biosynthetic genes and antioxidant properties of vitamin B6 vitamers during plant defense responses. *Physiological and Molecular Plant Pathology*.

[24] Morris MS, Picciano MF, Jacques PF, Selhub J (2008). Plasma pyridoxal 5′-phosphate in the US population: the National Health and Nutrition Examination Survey, 2003-2004. *American Journal of Clinical Nutrition*.

[25] Jackson LS, Al-Taher F (2005). Effects of consumer food preparation on acrylamide formation. *Advances in Experimental Medicine and Biology*.

[26] Zeeman SC, Thorneycroft D, Schupp N (2004). Plastidial *α*-glucan phosphorylase is not required for starch degradation in arabidopsis leaves but has a role in the tolerance of abiotic stress. *Plant Physiology*.

[27] Kossmann J, Lloyd J (2000). Understanding and influencing starch biochemistry. *Critical Reviews in Biochemistry and Molecular Biology*.

[28] Novy RG, Love SL, Corsini DL (2006). Defender: a high-yielding, processing potato cultivar with foliar and tuber resistance to late blight. *American Journal of Potato Research*.

[29] Novy RG, Whitworth JL, Stark JC (2008). Premier Russet: a dual-purpose, potato cultivar with significant resistance to low temperature sweetening during long-term storage. *American Journal of Potato Research*.

[30] Zommick DH, Knowles LO, Kumar GNM, Knowles NR (2011). Respiratory acclimation of tubers to temperature change reflects LTS responses of resistant/susceptible genotypes. *American Journal of Potato Research*.

[31] Blauer JM, Kumar GNM, Knowles LO, Dhingra A, Knowles NR (2013). Changes in ascorbate and associated gene expression during development and storage of potato tubers (*Solanum tuberosum* L. *Postharvest Biology and Technology*.

