# Supplementary material for: Genotype-Specific Changes in Vitamin B6 Content and the PDX Family in Potato
Source: Biomed Res Int. 2013 Jul 18;2013:389723. doi: 10.1155/2013/389723 (PMC3732595; doi:10.1155/2013/389723)
Supplement: Supplementary file 1 — Supplementary Table 1 summarizes findings for non-PDX proteins found in the performed Y2H screens on a mixed cDNA library containing samples from different potato tissues. The actual test for interactions of these proteins with PDX members is shown in Supplementary Figure 1. Major variation in total vitB6 content is present in fresh potato varieties purchased from a local supermarket (Supplementary Figure 2). Supplementary Table 2 provides an overview of primers used in this work. [file 389723.f1.docx]

| **Supplementary Table 1:** Yeast-2-Hybrid screen yield of interactors with | | |
| --- | --- | --- |
| **Bait AtPDX1.3** | | |
| Non-PDX clones found | | |
| *PGSC0003DMT400041662* | 2 | Histone superfamily similarity |
| *PGSC0003DMT400006347* | 4 | Phospholipid:diacylglycerol acyltransferase similarity |
| **Bait AtPDX2** | | |
| Non-PDX clones found | | |
| *PGSC0003DMT400046928* | 5 | No significant similarities to known proteins |
| *PGSC0003DMT400036050* | 3 | S-locus lectin protein kinase family similarity |

| **Supplementary Table 2:** Primers used in the Y2H screen, and for qRT-PCR analysis | |
| --- | --- |
| **Primer for qRT-PCR** | **5’-3’** |
| StPDX1.1-qF | CTGTGACTATTCCTGTAAT |
| StPDX1.1-qR | GTAATCTACTCCGATAGC |
| StPDX1.2-qF | TGCTCTAATCCTTACAAG |
| StPDX1.2-qR | GTAGGTCTCATCACTAAC |
| StPDX2-qF | ATTCCAATCCTGCTATTC |
| StPDX2-qR | CACAATATCAGAAGTTCCT |
| StActin7-qF | GGCTATGTATGTTGCTAT |
| StActin7-qR | ATCTTCATCAGGTTATCAG |
| **Primers for Y2H and colony screening** | |
| StPDX1.3-281FW | CAACTGGCCAGAAAATCAGT |
| StPDX1.3-281RW | GATCTGGTGATGCAAACAAAAC |
| StPDX1.3-2FW | CAGCTGGCCAGAAAATCAAA |
| StPDX1.3-2RW | CGATCTGGTGATGCAAACAAAG |
| St-clone 1-FW | GCACAATGGAACTTGGTTTATG |
| St-clone1-RW | GGACTTCTCCTTCTCACAAG |
| St-clone-7/10-FW | CTCTTTCAGCTTTGAGAGCTG |
| St-clone-7/10-RW | GTACTAATAGGTAGATGATTG |
| StPDX1-2R-FW | CCGGTACCGGAAACATCATC |
| StPDX1-2R-RW | GCTGGCCAGAAAATCAAAAC |
| StPDX1-281R-FW | CACCATACGATCTGGTGATG |
| StPDX1-281R-RW | CAGAGAGTATAACATACTCTC |
| StPDX1-168-1-FW | GCATTCTTTTGAACATATTC |
| StPDX1-168-1-RW | GTTcTTCAATGGGCACTTGATC |
| Stmth2-152-2-FW | CAGATCCATAGCATCGTGAG |
| Stmth2-152-2-RW | GATTAACCATGCGACCTGATC |
| S-1-FW | GTTTATGAAACAAAGAAAGC |
| S-1-RW | GATGGCAGGACTTCTCCTTC |
| S-2-FW | CATAGTTACACTACTTGATC |
| S-2-RW | GTCTACCTTCTTCCCACTTC |
| StPDX2FW | CTGGAAAGAAAGTAATTGTTG |
| StPDX2RW | GTTATAATGGTAGTACATAATG |
| S-3-FW | GCGGATACCTACAGCAGGATG |
| S-3-RW | GTGCACCACTCTGTGTACCAC |
| S-4-FW | GAGGACACCAATCTTTGTGCC |
| S-4-RW | GAACATTATCCCTACTATTAG |
