# Supplementary material for: Genotype-Specific Changes in Vitamin B6 Content and the PDX Family in Potato
Source: Biomed Res Int. 2013 Jul 18;2013:389723. doi: 10.1155/2013/389723 (PMC3732595; doi:10.1155/2013/389723)
Supplement: Supplementary file 2 [file 389723.f2.pdf]

Supplementary Figure 1

prey \ bait	pBTM:empty SDII	/	pBTM:empty SDIV	pBTM:AtPDX1.3 SDII	/	pBTM:AtPDX1.3 SDIV
PGSC0003DMT400041662	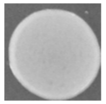	/	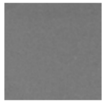	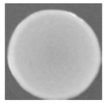	/	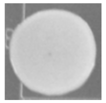
PGSC0003DMT400006347	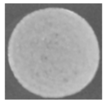	/	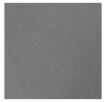	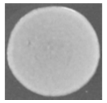	/	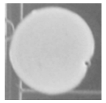

prey \ bait	pBTM:empty SDII	/	pBTM:empty SDIV	pBTM:AtPDX2 SDII	/	pBTM:AtPDX2 SDIV
PGSC0003DMT400046928	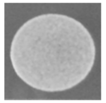	/	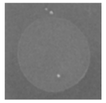	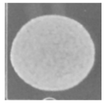	/	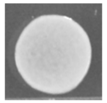
PGSC0003DMT400036050	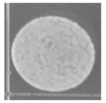	/	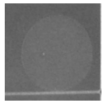	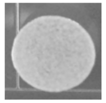	/	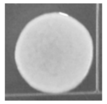
