# Supplementary figures and images for: Genotype-Specific Changes in Vitamin B6 Content and the PDX Family in Potato
Source: Biomed Res Int. 2013 Jul 18;2013:389723. doi: 10.1155/2013/389723 (PMC3732595; doi:10.1155/2013/389723)

Supplementary Figure 2

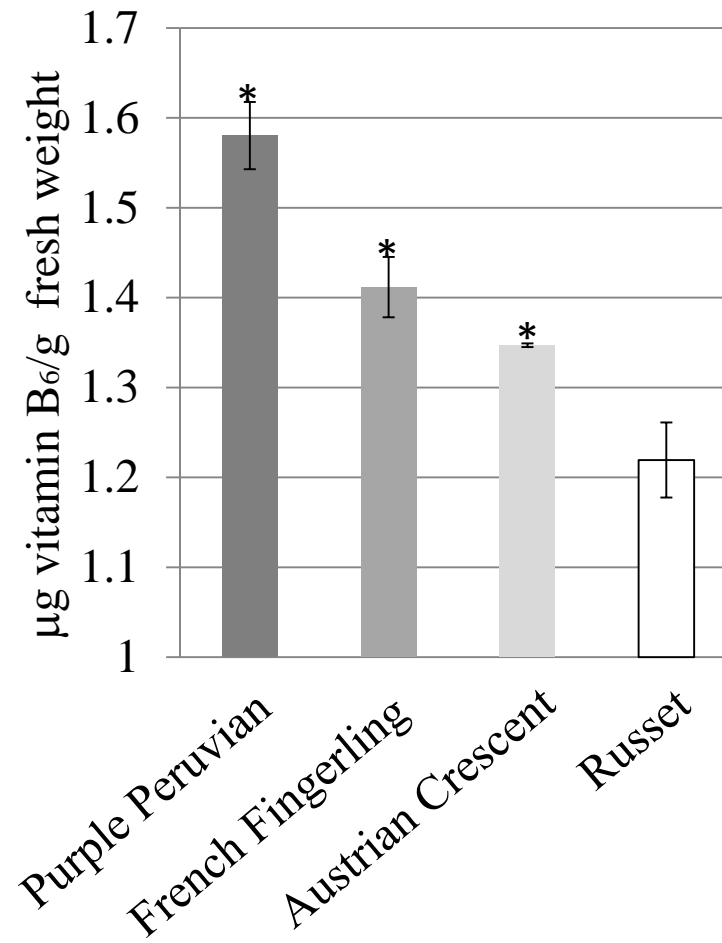

Supplement: Supplementary file 3 [file 389723.f3.pdf]
